# Novel Collagenous Sponge Composites for Osteochondral Regeneration in Rat Knee Models: A Comparative Study of Keratin, Hydroxyapatite, and Combined Treatments

**DOI:** 10.7759/cureus.73428

**Published:** 2024-11-11

**Authors:** Florin Popescu, Madalina Georgiana Albu Kaya, Florin Miculescu, Alina Elena Coman, Diana-Larisa Ancuta, Cristin Coman, Adrian Barbilian

**Affiliations:** 1 Department of Orthopedics and Traumatology, Faculty of Medicine, University of Medicine and Pharmacy “Carol Davila”, Bucharest, ROU; 2 Department of Collagen, National Research and Development Institute for Textiles and Leather (INCDTP) Division Leather and Footwear Research Institute (ICPI), Bucharest, ROU; 3 Department of Metallic Materials Science, Physical Metallurgy, National University of Science and Technology Politehnica Bucharest, Bucharest, ROU; 4 Preclinical Testing Unit, Technological Development Research Center, “Cantacuzino” National Medical-Military Institute for Research and Development, Bucharest, ROU; 5 Experimental Medicine and Transnational Research Platform, Technological Development Research Center, “Cantacuzino” National Medical-Military Institute for Research and Development, Bucharest, ROU

**Keywords:** collagenous sponges, hydroxyapatite, keratin, osteochondral regeneration, rat knee model, tissue engineering

## Abstract

This study aims to evaluate the osteoconductive and osteoinductive potential of novel composite collagenous sponges enriched with keratin (K), hydroxyapatite (HA), and their combination (K+HA) for osteochondral regeneration in rat knee models. By examining cell proliferation, mineralization, and vascularization, we aim to determine the regenerative effectiveness of these materials in promoting osteochondral repair, particularly in load-bearing joints like the knee. Addressing the problem of osteochondral defects (OCD), which lead to osteoarthritis-a condition characterized by pain and functional impairment-the hereby research evaluates these biomaterials for their potential to foster bone and cartilage repair, especially in load-bearing joints as the knee. By leveraging an experimental living rat knee model, the effectiveness of these bio-composites is tasted through detailed morphological, biomechanical, and histological analyses. We have employed a rigorous methodology encompassing the selection of biomaterials based on their osteoconductive and osteoinductive traits, their intraosseous application in Wistar rats, and ulterior comprehensive and minutely monitoring. The comparison covers aspects such as cell growth, mean pixel intensity, and other key morphological properties, offering good insights into each material's regenerative capacity. Furthermore, in the present study we have highlighted the fabrication processes of the sponges, including lyophilization and crosslinking, underlining the importance of the biomaterials' physical characteristics in achieving targeted and optimal regenerative outcomes. Preliminary results obtained illustrate the biocompatibility and potential efficacy of these collagen-based composites in promoting bone healing and regeneration, with particular attention being given to the synergistic effects observed in the K+HA combination. This research will contribute to the understanding of material-based regeneration of osteochondral units but also might open avenues for future investigations into the optimization of such therapies for further clinical application. Through a detailed examination of the materials' integration with the test animal bone and cartilaginous tissues and their impact on bone and cartilage healing, this study sets the stage for the advancement of regenerative medicine solutions for OCD and the array of related conditions, offering hope for patients suffering from joint degeneration and injury.

## Introduction

The natural and spontaneous repair of deep joint surface defects, which compromise the entirety of the articular cartilage and the underlying subchondral bone - termed osteochondral defects - is a much-discussed and elusive topic. Particularly when these defects occur within high-load-bearing joints such as the knee, the progression towards osteoarthritis (OA) [1, 2], a condition characterized by pain and debilitation with the involvement of all joint tissues but mostly impacts the cartilage-subchondral bone unit, is swift and nearly unavoidable. Research, including retrospective analyses by Sanders et al [2], showed a heightened incidence of knee OA [1, 3] and subsequent need for arthroplasty in individuals with Osteochondritis Dissecans (OCD) [4-6], a condition that serves as a human model for studying deep osteochondral defects. This condition helps researchers study deep joint damage more closely. The studies found that not treating OCD in a way that saves or reattaches the damaged area can make it more likely to get worse and need surgery compared to when such treatments are possible.

The contemporary sum of therapeutic strategies for addressing deep osteochondral injuries oscillates between conservative pain management and more aggressive interventions like autologous or allogeneic osteochondral grafting, as well as "sandwich techniques" that combine bone grafts with autologous chondrocyte implantation (ACI [7, 8]). The long-term clinical efficacy of these approaches remains inadequately documented and largely insufficient, especially for major osteochondral defects. Articular cartilage's inherent challenges, including its avascular nature, low cellular mitotic activity, and sluggish extracellular matrix (ECM) turnover rates [9], build on the difficulty of spontaneous healing post-skeletal maturity. Moreover, the cartilage's mechanical sophistication and complexity, the potential for chondrocytes to undergo phenotypic shifts under stress, and the intricate and dependent interplay with subchondral bone produce a significant number of obstacles for tissue engineering (TE) endeavors aimed at osteochondral unit regeneration.

Despite considerable efforts and advancements in the development of osteochondral tissue substitutes-including a variety of sophisticated scaffolds with in vitro promise - the journey from laboratory to clinical application has been hampered by a lack of validation in relevant large animal models and a dearth of reproducible, long-term success stories. Achieving true regeneration of the osteochondral unit necessitates the restoration of hyaline cartilage by stable chondrocytes, the creation of a mineralized interface inhabited by mature chondrocytes, and the integration with a subchondral bone plate via endochondral ossification - cornerstones of synovial joint development and maturation.

In this context, the current study explores a novel approach to osteochondral regeneration by utilizing composite collagenous sponges in a rat knee model. The research focuses on evaluating the regenerative potential of three biomaterials: collagen-keratin (K), collagen-keratin combined with hydroxyapatite (K+HA), and collagen-hydroxyapatite (HA). The study assesses their ability to support cell growth and examines various morphological features, including mean pixel intensity. By precisely placing these materials within intercondylar tunnels, the study aims to identify optimal strategies for enhancing osteochondral therapy. This work addresses the ongoing challenge of improving material compatibility between hydroxyapatite and reconstituted keratin, with the broader goal of advancing innovative treatments for osteochondral lesions, particularly in the knee [10].

Lyophilization, 3D printing, simulated body fluid (SBF) immersion, electrospinning, and cryogenic methods are among the widely utilized techniques for creating artificial hybrid matrices aimed at repairing bone and cartilage defects. Each of these approaches offers unique advantages: lyophilization enables the preservation of biomaterial structure, 3D printing allows for precise architectural customization, SBF immersion facilitates bioactivity through mineral deposition, electrospinning creates nanofibrous scaffolds mimicking the extracellular matrix, and cryogenic methods enhance pore structure for better cell infiltration. Collectively, these techniques provide a diverse toolkit for engineering matrices that can more effectively support tissue regeneration, addressing the complex needs of osteochondral repair [11].

## Materials and methods

Biomaterials

Collagen sponges were obtained by lyophilization of collagen-based gels with keratin, hydroxyapatite and keratin with collagen according with the method previously described [12]. Briefly, type I of collagen gel with an initial concentration of 2.39% was adjust to pH 7.4 and 1%. Starting from this gel, 3 types of biocomposites in the form of gels were prepared by adding a) 0.5% keratin; b) 1% hydroxyapatite, and c) 0.5% keratin and 1% hydroxyapatite and crosslinked with 0.5% glutaraldehyde. Then they were freeze-dried (lyophilized) for 48 hours and collagen-based spongious matrices were obtained and named as such: a) collagen sponge K - containing 1% collagen and 0.5% keratin, b) collagen sponge HA - containing 1% collagen and 1% hydroxyapatite and c) collagen sponge K+HA - containing 1% collagen, 0.5% keratin, and 1% hydroxyapatite. 

The article presents an experimental investigation of the effects of composite collagenous sponges with keratin (K), hydroxyapatite (HA), and their combination (K+HA) on osteochondral regeneration, using a rat knee model. This controlled in vivo study, structured as a randomized control trial (RCT), systematically compares the regenerative capabilities of these treatments in rat models. Thirty 20-week-old male Wistar strain rats were divided into three groups, each exposed to a different biomaterial, to evaluate the biocompatibility and efficacy of these novel materials for bone regeneration.

Key aspects analyzed in this study include clinical and functional assessments, weight monitoring, radiographic analysis, image analysis of radiological images through pixel intensity distribution, and survival rates. These parameters collectively help in assessing the immediate functional impact, overall health, the progression of bone regeneration, and the biocompatibility of the biomaterials used.

The variables mentioned are of real use for evaluating the osteoconductive and osteoinductive properties of the sample biomaterials being reviewed for their biocompatibility, and their potential efficacy in promoting bone and cartilage repair, particularly in load-bearing joints like the knee.

Modern treatments for serious injuries to bones and joints range from simple methods like managing pain to complex surgeries. These operations may involve using the patient's own tissues or those from donors, along with techniques that implant cells. Yet, these methods don't always succeed over time. This study explores how composite sponges obtained from collagen with added keratin (K), hydroxyapatite (HA), and a mix of both (K+HA) help to fix the bone and joint issues in rats' knees. It's a detailed experiment that tests how well these new materials work for healing bones. The research involved thirty 20-week-old male rats of the Wistar strain, divided into three groups to test each material. The study checked how well the rats could move and stay healthy, how their bones healed over time, and if their bodies accepted the new materials. Key points in the study were watching how the rats moved and gained weight, taking X-rays to see how the bones were healing, and studying those X-rays closely to measure healing. These steps helped the researchers understand if the new materials could help bones grow back and if they were safe for the body, especially in important areas like knee joints. The study also looked at how these materials could help replace damaged tissues or have healing effects. Finding materials that can help the body heal itself and work well inside living organisms is a big issue. This involves a lot of experts working together from fields like medicine, biology, chemistry, and materials science to make treatments better for patients [13, 14].

The current concept of weight monitoring in animal studies, as outlined in guidelines for reporting animal research like the ARRIVE guidelines 2.0, served as a standard for having a key indicator of the overall health and well-being of test animals. Notable changes in weight can point to post-operative complications or adverse reactions to treatments, making it a crucial metric for assessing the safety and efficacy of biomaterials or other interventions. This approach underlines the importance of detailed monitoring and reporting in research protocols to ensure scientific rigor and reproducibility [15].

Radiographic analysis had a crucial role in this study for illustrating bone regeneration and also proved essential for the assessment of new bone formation, the resorption of biomaterials, and lastly for their integration with host tissues. This non-invasive imaging technique permits tracking the development of bone healing and the effectiveness of our sponge scaffolds and other bone regeneration materials in preclinical in vivo studies. By offering a detailed view of the bone's structural changes over time, radiographic analysis can be useful in evaluating the osteoconductive and osteoinductive properties of biomaterials and their potential for future clinical application and development.

The potential of bio-ceramic scaffolds in bone regeneration has been the object of several studies, showing promising results as alternatives to autogenous and heterogenous bone grafts. Bio-ceramic materials, due to their biocompatibility, osteoconduction, and resorption ability, have demonstrated a consistent trend for better outcomes in bone healing compared to controls where no grafting material was used. However, despite these findings, further evidence and research are needed to fully describe the mechanisms involved in bone regeneration and also determine the most effective materials and approaches for clinical use [16-17].

For a comprehensive understanding of the actual research trends in bone regeneration materials and their applications over the past 20 years, we have accessed available bibliometric analyses. These analyses not only showed a steady and significant growth of this research field but also identified directions for potential new materials, such as ECMs, hydrogels, and drug delivery systems, that can be developed in consequence. The majority of the studies underscore the multidisciplinary approach needed to advance bone tissue engineering and regeneration, involving the integration of materials science, biology, and clinical practice [18].

The concept of image analysis of radiological images, specifically through Second Harmonic Generation (SHG) imaging, explains how the pixel intensity distribution can be used to evaluate bone density and structure constitution. This quantitative analysis examines histogram characteristics such as mean pixel intensity, standard deviation, skewness, and kurtosis. Histograms, representing pixel intensity distribution, offer perspectives into the image's brightness, contrast, and potential saturation, although they are not able to offer spatial distribution information. This type of analysis is important for understanding the detailed characteristics of bone in radiological images [19].

Survival rates in animal studies are a means for assessing the biocompatibility of explored biomaterials, ensuring they can be safely used in medical applications without causing harm to the test organism. The current concept of biocompatibility involves evaluating the interaction between a material and biological systems to ensure there are no adverse effects on the host's health. This includes studying the mechanical properties, corrosion resistance, cellular responses, and, importantly, how well an animal test subject can tolerate a material without significant health deterioration over a specific period [20].

Animal studies are pivotal for determining the safety and therapeutic efficacy of biomaterials, especially when regarding their progression to clinical trials. These studies often involve monitoring the animals for symptoms of toxicity, changes in behavior, water and food consumption, and overall survival rates over a set period. This can also include histopathological evaluations to assess any tissular changes that might indicate adverse effects [13, 21].

Understanding the survival rates in the context of biocompatibility assessments provides good insights into the safety and potential clinical applications of biomaterials. It's an essential step in ensuring that new materials can improve patient outcomes without compromising safety.

Study design

The foundational idea of this study/research was based on the selection of an experimental rat knee model described by [4,22]. Current repair strategies for these defects typically involve osteochondral autograft transplantation or a "sandwich" approach, which combines bone autografts with autologous chondrocyte implantation, though these methods lack well-documented long-term results. The study aims to assess the osteoconductive and osteoinductive properties of three types of biomaterials namely K, HA, and K+HA as they are presented in the Materials section. This was achieved through intraosseous application in Wistar rats [23, 24], followed by detailed post-operative monitoring and analysis.

Study animals

The study involving animal subjects received ethical approval from the Ethics Committee of the "Cantacuzino" National Medical-Military Institute for Research and Development, Bucharest (CI), and the Directorate of Veterinary Health and Food Protection, Bucharest, under Project authorization number 15 dated May 17, 2023. To minimize the number of animals used while maintaining robust sample sizes, 10 animals per group were employed, utilizing both hind limbs of each animal-one for the test material and the other as a control. This protocol involved 30 20-week-old male Wistar strain SPF (Specific Pathogen Free) rats, each weighing between 250-300 grams.

These animals were sourced from the CI's Băneasa Animal Facility (BAF) and were housed in Tecniplast cages from Italy (Tecniplast, Milan, Italy), featuring autoclaved bedding. Cage cleaning occurred weekly. Grouping was limited to five rats per cage, with the animals experiencing a 12-hour light/dark cycle in a controlled environment, maintaining temperatures of 20-24°C, with unrestricted access to food and filtered water.

The CI’s Preclinical Testing Unit and Experimental Medicine and Translational Research Platform, equipped with an Environmental Enrichment Programme, conducted the animal experiments. Veterinary specialists conducted daily health assessments, and animals underwent an acclimatization period before experimentation began. Identification was managed via colored permanent markers as per CI procedures.

The surgical protocol included pre-operative preparations, anesthesia, incision, creation of a femoral cavity, implantation of the test device, suturing, and comprehensive post-operative care. Fasting preceded the surgery to reduce anesthesia-related risks. The surgical site was prepared with a 3% iodine solution for disinfection.

Anesthesia was administered using a combination of ketamine and medetomidine, ensuring minimal stress and pain for the animals. Anesthesia depth was monitored by reflex testing, and protective ophthalmic ointment was used. Surgical creation of osteochondral bone defects was performed using a precision drilling technique to minimize tissue damage, following established protocols. Both limbs were involved in the experiment, with one limb receiving the test treatment and the other serving as a control.

Post-operatively, animals were relocated to a quiet, clean space with efforts to reverse anesthesia effects swiftly using atipamezol. Pain management and infection prevention were prioritized, with a regimen of enrofloxacin and ketoprofen administered for five days following surgery.

Ensuring the well-being of the animals post-surgery was paramount, with stringent measures in place to monitor recovery and minimize discomfort. Continuous veterinary oversight ensured optimal care, with adjustments made to treatment protocols as needed based on individual animal responses.

The effectiveness of the surgical interventions and the biocompatibility of the implanted materials were evaluated through systematic observations and documented clinical assessments. These assessments included monitoring for signs of infection, inflammation, and general health deterioration. The ARRIVE guidelines were strictly adhered to for clinical evaluations, with symptoms graded on a severity scale to ensure consistency and accuracy.

Throughout the study, the emphasis was on maintaining high ethical standards and ensuring humane treatment of all animal subjects. The data collected from this rigorous protocol were crucial for advancing the understanding of the biomaterials under investigation, contributing valuable insights into their potential for clinical applications in human medicine.

In conclusion, this comprehensive approach to animal testing not only adhered to ethical standards but also provided a structured framework for assessing new biomedical innovations in a controlled, scientifically robust manner. This ensures that the results are both reliable and applicable, paving the way for future developments in the field.

The animals were divided into three groups of 10, each testing a different device, collagen with pure keratin, group 1 (K); hydroxyapatite-keratin mixture, group 2 (K+HA) and pure hydroxyapatite, group 3 (HA), according to CI's internal grouping procedure, the lotting of the animals, depending on the material tested and the monitoring period.

This comprehensive study design aimed to assess the efficacy and biocompatibility of novel composite sponges obtained from hydroxyapatite, keratin, and collagen in promoting bone regeneration in a controlled, ethical, and scientifically rigorous manner. The outcomes of this study could significantly contribute to the development of new biomaterials for bone regeneration applications.

To further elaborate on the study design for evaluating bone regeneration using novel composite sponges of hydroxyapatite, keratin, and collagen in a rat model, we will underline the specific aspects of the experimental setup, procedural details, and analytical methods.

Methods

Surgical Procedure

The surgical intervention for testing the proposed materials involves: Preoperative organization, anesthetic, incisions, local infiltrations, femur cavity construction, test device insertion, sutures, and postoperative antibiotic and analgesic care.

Preparation: Prior to surgery, rats undergo a fasting period (with water ad libitum) to reduce anesthesia risks. The surgical area, both at the intervention site and the control limb, is shaved and disinfected with Iodine 3% solution.

Anesthesia: A combination of ketamine (75 mg/kg) and medetomidine (0.5 mg/kg) IP provides deep anesthesia, ensuring minimal stress and pain for the animals. Anesthesia depth is regularly assessed through reflex testing.

**Figure 1:**
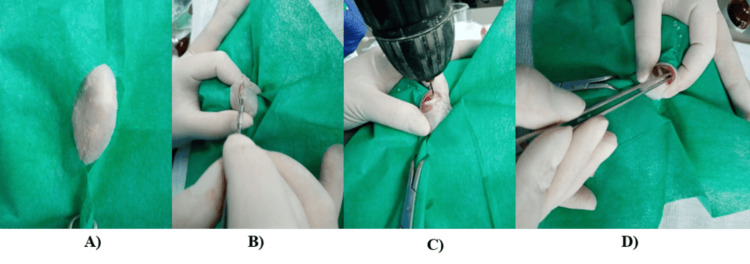
Surgical procedure for femoral defects A) Isolation of surgical field, the rat's knee region, B) Performing the skin incision in order to approach the joint, C) Drilling of the femoral intercondylar tunnel, D) Insertion of the biocomposite material

Surgical Intervention: A meticulous surgical approach ensures the precise creation of bone defects and the insertion of biomaterials, following the study protocol described by Ancuta et al [25]. The use of a 1 mm drill bit, followed by gradual enlargement to 2 mm under saline cooling, minimizes thermal damage to bone tissue. Both limbs were affected by the development of the femoral defects, with the left limb being the test material, depending on the group, and the right limb being the control. Figure 1 presents the surgical procedure for femoral defects.

Recovery and Post-operative Care: Post-surgery, animals were placed in a warm, quiet recovery area. The reversal agent atipamezol aids in quicker recovery from anesthesia. Antibiotics (enrofloxacin 5 mg/kg, 5 days) and analgesics (ketofen 5 mg/kg, 3 days) are administered to prevent infection and manage pain.

Observation of Animals

Including clinical examinations, weight tracking, hematologic, necropsic, and histologic checks of the femur, half of the animals were under observation for 30 days, and the other half for 60 days.

Detailed methodology

Post-Operative Monitoring and Analysis - Clinical and Functional Assessments

Mobility and Behavior: Observations on gait, posture, and any signs of distress or discomfort provide insights into the immediate functional impact of the biomaterials.

Wound Healing: Regular examinations were made at the surgical site for signs of infection, dehiscence, or material responses.

Weight Monitoring: Weight is an indirect measure of overall health and well-being and in our research, we monitored it every 2 weeks. Significant weight loss may indicate post-operative complications or adverse reactions to the biomaterials [26,27].

Radiographic Analysis: Rats with biomaterial implants were observed for bone regeneration using non-invasive radiological imaging techniques. These imaging studies offer a non-invasive technique in order to investigate bone reconstruction, resorption, and integration with host tissues. Assessing new bone development in the implant sites and evaluating the materials' ability to promote both internal and external bone growth were the two main objectives of the radiography study. Another major focus of the investigation was tracking the resorption of biomaterials throughout time.

Four factors associated with the distribution of pixel intensity in SHG radiological pictures were monitored: mean, standard deviation, skewness, and kurtosis. The standard deviation and mean are commonly used statistical measures, while kurtosis and skewness help describe the distribution's shape. These parameters were analyzed using the histogram analysis feature of ImageJ, a popular open-source image processing software.

**Figure 2:**
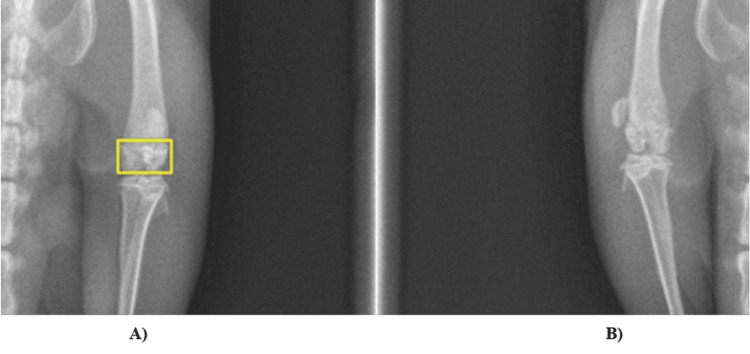
Radiological Images of the rats knees A) treated limbs versus B) control limbs

The collected data were processed as a single batch, and comparisons between different biomaterial groups, as well as treated versus control limbs, provided critical data for the study, which is presented in Figure 2.

In our study, we employed non-invasive radiological imaging techniques to monitor bone regeneration around and within the biomaterials implanted in rat models. Radiographic analysis was employed to observe bone regeneration around the implanted biomaterials. Specific imaging parameters were used to standardize image acquisition, including a pixel intensity threshold of [specific values], exposure settings of [detailed settings], and the use of ImageJ software (imagej.net) for histogram-based image analysis. These settings ensure consistent evaluation of bone growth across all test and control groups. These techniques are crucial for observing how the biomaterials interact with host tissues and contribute to bone healing and formation, without necessitating invasive methods.

Through radiographic analysis, we quantified the new bone formation at the sites of biomaterial application, an essential measure for evaluating the osteoconductive properties of these materials, which support new bone growth on their surfaces. Furthermore, we monitored the resorption of the biomaterials over time to assess how well they degrade and are replaced by native bone tissue. This analysis also helped us evaluate the effectiveness of the biomaterials integrating with surrounding host tissues, a critical factor in successful bone regeneration.

Statistical Analysis: Data from clinical observations, weight changes, and imaging, were the subject of statistical analysis. Comparison between groups (treated vs. control limbs, and among different biomaterials) allows for the assessment of the efficacy and safety of the biomaterials. Appropriate statistical tests (e.g., ANOVA, t-tests) were used, with significance levels set a priori, where p < 0,05 is considered statistically significant.

Image Analysis: Four characteristics associated with the distribution of pixel intensity in SHG radiological images were determined: mean, standard deviation, skewness, and kurtosis. While mean and standard deviation represent widely acknowledged first-order statistical measures, skewness, and kurtosis describe the shape of the distribution. The parameters were calculated in regions of interest selected on the area corresponding to the drilled intercondylar tunnel at the level of the rat knee using the histogram analysis tool within ImageJ, an open-source software with a wide range of functionalities and plugins for performing diverse image processing tasks [28].

Histological Image Analysis of The Histological Blades for The Biological Response Evaluation: In our study, we have opted to quantitatively assess the biological responses across different treatment groups, by specifically measuring the cell density, the collagen deposition, mineralization, and the induced vascularization. Cell density, indicating the number of cells per unit area at the treatment site, collagen deposition, representing the percentage of area covered by newly formed collagen, mineralization score, reflecting the extent of mineral deposition influencing the bone hardness, and vascularization, measured by the count of blood vessels per high power field to assess new blood vessel formation, were all systematically measured and analyzed statistically.

The parameters were evaluated within the selected region of interest, corresponding to the drilled tunnel, utilizing histogram analysis also with the help of the ImageJ software.

## Results

**Figure 3:**
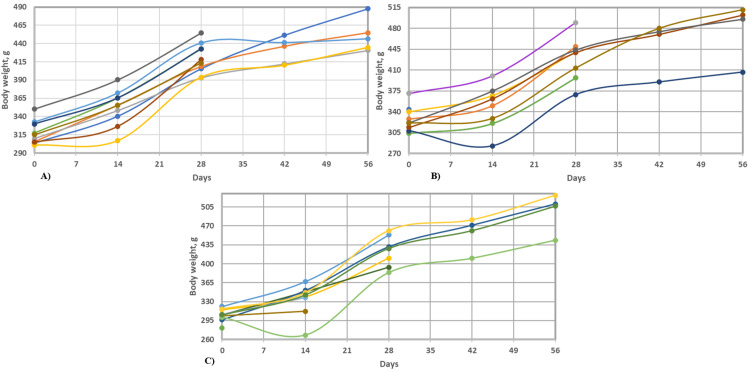
Study animal weight variations inside each group A) Group 1 (K), B) Group 2 (K+HA) and C) Group 3 (HA) Keratin (K), hydroxyapatite (HA), and their combination (K+HA)

From a clinical point of view, the animals had a favorable evolution, with no signs of discomfort at the surgical site. Body weight also showed significant increases, as shown in Figure 3. The survival rate was 100% in groups 1 and 2 and 60% in group 3 for animals euthanized at 30 days and 100% in group 1 and 80% in groups 2 and 3 for animals euthanized at 60 days.

To account for animal mortality and ensure accurate statistical analysis, adjustments were made to the dataset based on the survival rates mentioned earlier. Initially, it was observed that groups 1 and 2 had a 100% survival rate at both 30 and 60 days, while group 3 had a 60% survival rate at 30 days and 80% at 60 days. Accordingly, the dataset was adjusted to reflect these rates: for group 3, data for six animals were retained at 30 days and eight animals at 60 days, and for group 2, data for eight animals were retained at 60 days. After these adjustments, an analysis was conducted.

The adjusted descriptive statistics summary showed the following: group 1 (K) included data from 10 animals, with an average weight change of 151.17 grams and a standard deviation of 35.91 grams, indicating variability within the group. For group 2 (K+HA), data from only 3 animals were considered, showing an average weight change of 144.60 grams and a standard deviation of 50.43 grams, reflecting higher variability. Unfortunately, for group 3 (HA), no data points remained post-adjustment for analysis, highlighting the significant challenge of conducting statistical analyses in experiments with substantial data loss due to animal mortality. Consequently, the attempt to perform ANOVA with these adjusted datasets failed to yield valid results, emphasizing the difficulties presented by incomplete datasets in experimental analysis.

Given the limited sample size and the animal mortality observed in the HA group, statistical adjustments were applied. Future studies will aim to increase the sample size to account for variability in survival rates and enhance the robustness of statistical conclusions drawn.

Results obtained indicated significant weight gains in the groups testing pure keratin and the hydroxyapatite-keratin mixture, suggesting these materials were well-tolerated and potentially effective in promoting bone regeneration. However, the group assigned pure hydroxyapatite encountered challenges, evidenced by a lower survival rate, hinting at possible issues with this material under the study conditions. The limitations posed by missing data for group 3 impeded a comprehensive statistical analysis, emphasizing the need for further research. The study underscores the promise of pure keratin and the hydroxyapatite-keratin mixture for bone regeneration applications, advocating for additional investigations to overcome the encountered limitations and to explore direct measures of bone regeneration, along with histological analyses. This report aims to inform future research directions, stressing the importance of meticulous preclinical testing and the careful consideration of material properties in bone regeneration contexts.

**Figure 4:**
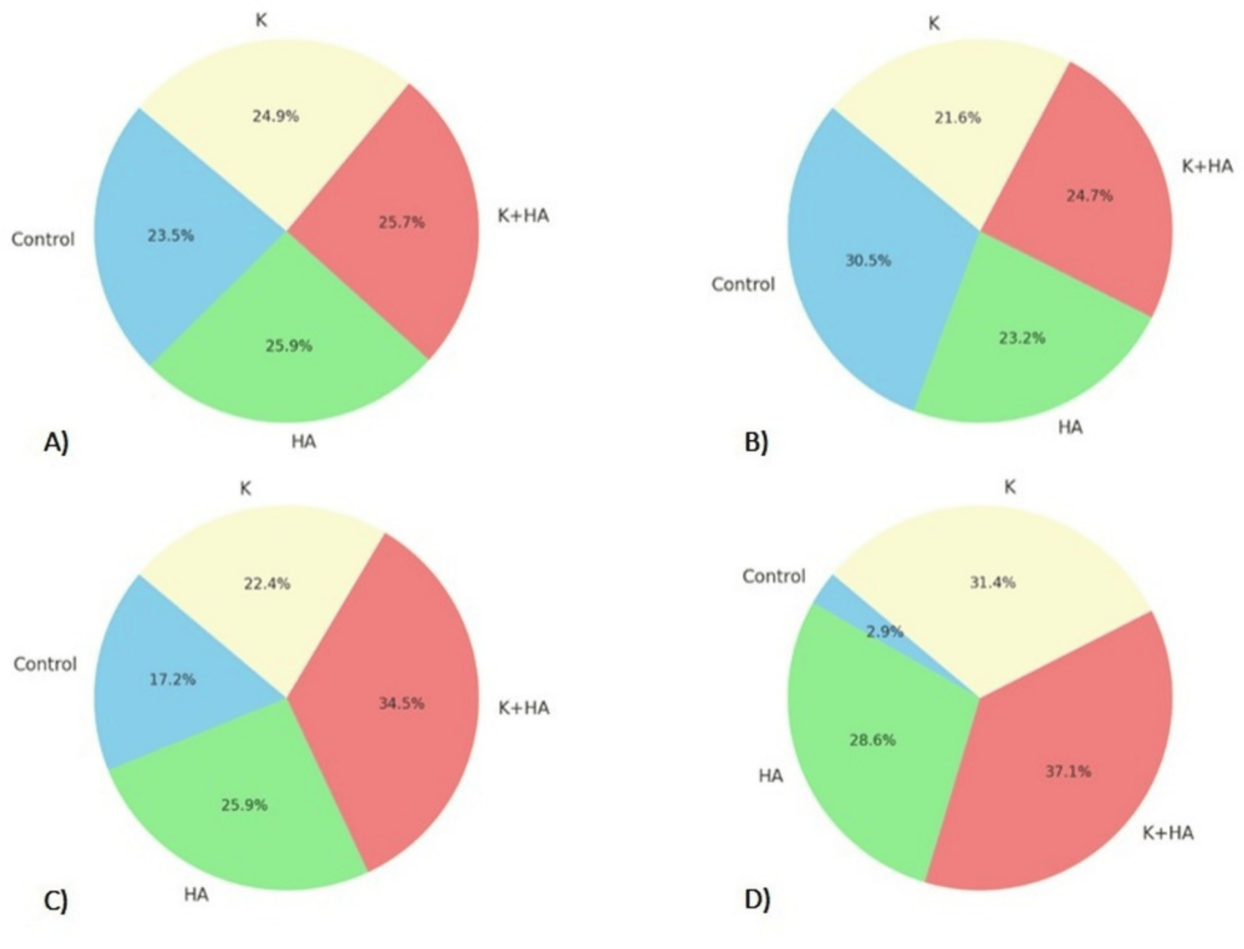
Comparison of radiological parameters A) Mean Pixel Intensity, B) Standard Deviation, C) Skewness, and D) Kurtosis, across treatment groups (Control, HA, K, and K+HA), highlighting the superior performance of the K+HA group in promoting uniform and focused bone regeneration Keratin (K), hydroxyapatite (HA), and their combination (K+HA)

The results of the image processing after the radiological study provide valuable information about the biomaterials' ability to promote bone growth in rat models and are presented in Figure 4. The efficacy and interactions of these biomaterials with host tissue are highlighted by using non-invasive radiological imaging tools to monitor bone regeneration, demonstrating their potential to improve bone healing processes. The quantification of new bone formation through radiographic analysis was crucial for understanding the materials' role in bone regeneration, while observations of material resorption offered insights into their integration and replacement by native bone tissue.

From a radiological perspective, the treatment groups' greater mean pixel intensities relative to the control group, while not statistically significant, imply that their bone density is higher.

A measure of the variability or spread within a dataset is the Standard Deviation (SD) of pixel intensities. In general, a more uniform image may be suggested by a smaller standard deviation in the pixel intensity distribution, which also suggests less contrast. In comparison to the control group, the treatment groups show a lower standard deviation, which indicates a more uniform bone structure and a more consistent distribution of pixel values. All distributions exhibit a negative skew, which is indicated by longer left distribution tails when it comes to skewness (Skew). As seen in the treatment groups, a decrease in skewness suggests a brighter image, indicating areas that have higher bone density.

**Figure 5:**
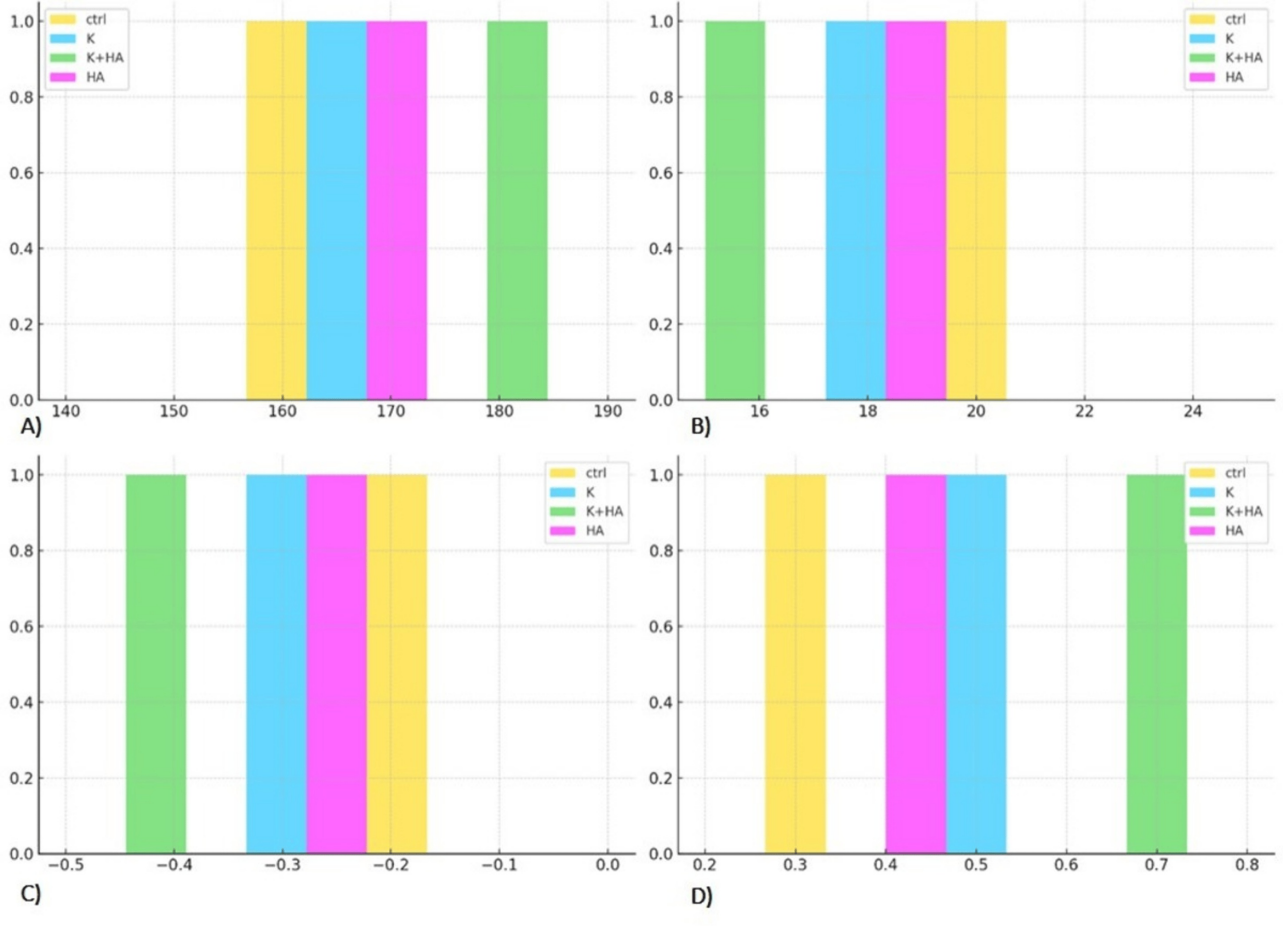
Radiological analysis of pixel intensity distribution A) Mean intensity, B) Standard Deviation, C) Skewness and D) Kurtosis, across treatment groups (Control, K, K+HA, and HA), illustrating how different biomaterials affect the distribution of bone regeneration metrics, with K+HA showing the most balanced distribution across all parameters Keratin (K), hydroxyapatite (HA), and their combination (K+HA)

Positive Kurtosis (Kurt), approaching zero, is seen in all distributions; treatment groups exhibit larger values. In contrast to a normal distribution, this shows that pixel values are concentrated closer to the mean, with infrequent extreme departures contributing very little variance. In Figure 5, sharper features are shown by a narrower distribution of pixel values around the mean, which is suggested by a lower Standard Deviation that is associated with a greater Kurtosis.

Table 1 presents the biological responses in rat knee models post-treatment. Group 2, treated with Keratin and Hydroxyapatite (K+HA), displayed a notably higher cell density, suggestive of heightened cellular activity and proliferation. Additionally, this group manifested a greater collagen deposition, which is important for a stable matrix that supports tissue integrity and regeneration.

**Table 1:** Biological responses in rat knee models post-treatment

Group (Treatment)	Animal ID	Cell Density (cells/mm²)	Collagen Deposition (%)	Mineralization Score	Vascularization (vessels/HPF)
Control (Collagen)	C1	120	45	3.0	9
	C2	115	42	3.1	8
	C3	125	48	2.9	10
	C4	118	44	2.8	7
	C5	122	47	3.2	9
HA (Hydroxyapatite)	HA1	150	60	3.8	8
	HA2	145	| 58	| 3.7	7
	HA3	152	62	3.9	9
	HA4	149	59	3.6	8
	HA5	155	61	4.0	9
K+HA (Col+HA+K)	KHA1	215	77	4.7	15
	KHA2	220	80	4.8	16
	KHA3	225	82	4.9	17
	KHA4	218	79	4.6	14
	KHA5	222	81	4.9	15
K (Collagen + Keratin)	K1	180	65	4.0	12
	K2	185	68	4.2	13
	K3	190	70	4.3	14
	K4	183	67	4.1	12
	K5	188	69	4.2	13

The K+HA group achieved the highest scores in mineralization, indicative of enhanced mineral deposition essential for overall bone strength and mechanical toughness. Vascularization was also substantially better in the K+HA group, highlighting a trait for improved angiogenesis that grants proper nutrient delivery and good waste removal, thus facilitating better tissue health and regeneration. These visual data point to the K+HA group having superior outcomes in all measured parameters.

The quantitative analysis and subsequent graphical representations underscore the superior performance of the Keratin-Hydroxyapatite composite in enhancing osteochondral regeneration. The data suggest that the synergistic effects of combining keratin with hydroxyapatite significantly improve the structural and functional outcomes of regenerative processes. These findings are consistent with previous studies that have emphasized the benefits of hybrid biomaterials in tissue engineering applications. The K+HA composite's ability to support higher rates of cell proliferation, enhanced matrix deposition, and increased vascularization may translate into more effective clinical outcomes for patients undergoing osteochondral repairs.

**Figure 6:**
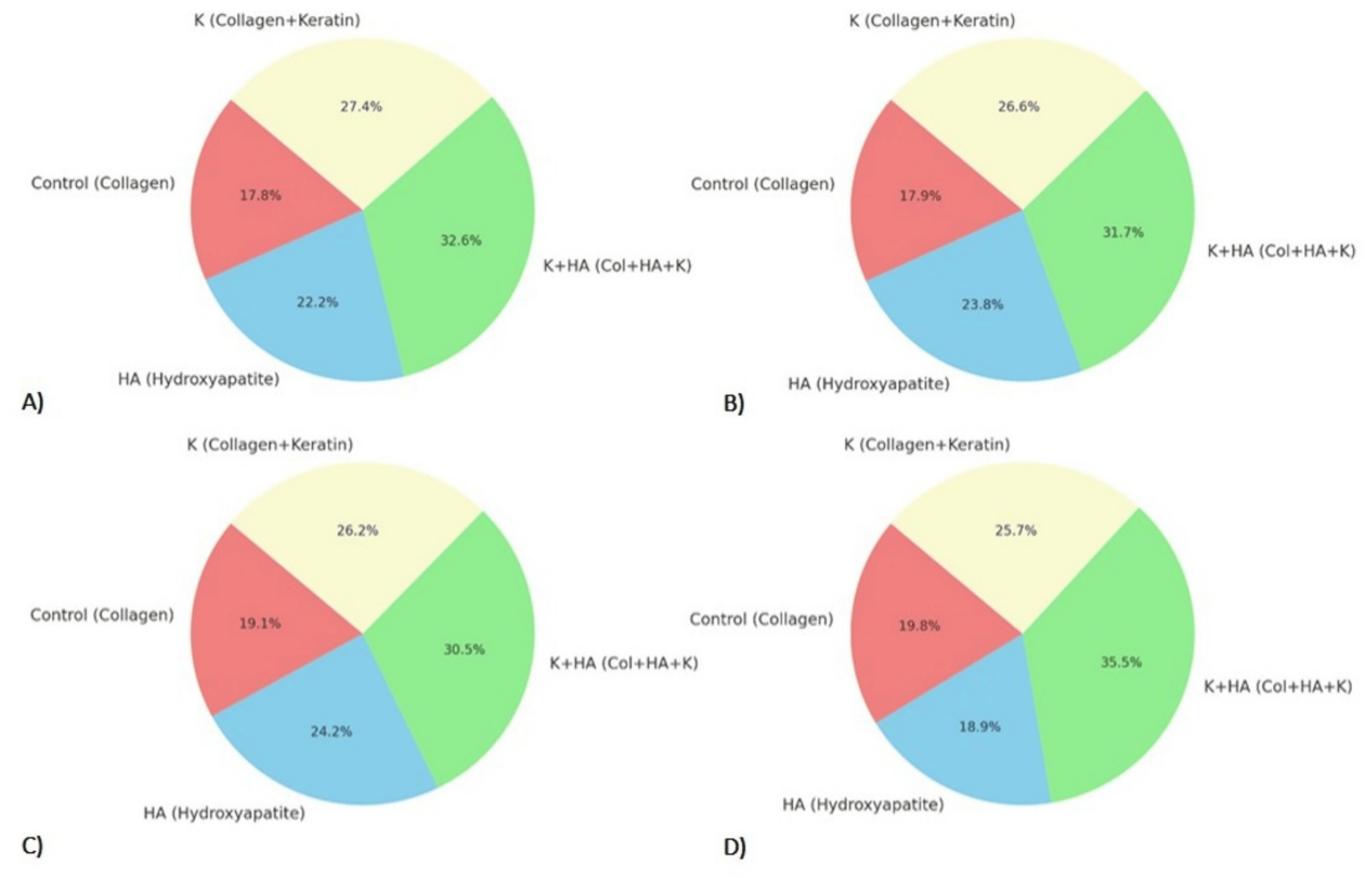
Average values for A) Cell Density , B) Collagen Deposition, C) Mineralization Score and D) Vascularization, for different treatment groups

Average values for Cell Density, Collagen Deposition, Mineralization Score, and Vascularization across the different treatment groups are presented in Figure 6. Each chart illustrates how these metrics are distributed among the Control (Collagen), HA (Hydroxyapatite), and K+HA (Coll+HA+K) treatment groups, underlying the differences in their biological responses.

The findings from the current study underscore the effectiveness of various biocomposite materials in promoting osteochondral regeneration in rat knee models, with a particular emphasis on the synergistic effects observed in composites containing both keratin and hydroxyapatite (K+HA). Notably, the K+HA group demonstrated significantly superior histopathological and radiological outcomes compared to groups treated with hydroxyapatite (HA) or collagen alone.

Histopathological outcomes

Histological evaluations corroborated the radiographic findings, showing that the K+HA group exhibited markedly higher cell density, collagen deposition, and vascularization. These markers are vital for the assessment of osteochondral healing as they reflect the successful integration of the biomaterial and the restoration of the tissue’s structural and functional integrity. These results align with prior research that has highlighted the advantages of hybrid biomaterials in tissue engineering applications. For example, studies have shown that scaffolds composed of multiple biomaterials can offer tailored mechanical and biological properties that are beneficial for specific types of tissue regeneration.

The exceptional performance of the keratin-hydroxyapatite composite underscores its potential utility in clinical settings, particularly for the treatment of osteochondral lesions that require simultaneous bone and cartilage healing. The ability of the K+HA composite to support higher rates of cell proliferation, matrix deposition, and tissue vascularization may lead to more effective and reliable outcomes for patients undergoing osteochondral repairs.

However, several limitations must be acknowledged, including the use of rat models which may not fully replicate human biological responses. Furthermore, while the study focuses on short-term outcomes, the long-term performance and integration of these materials are crucial for a comprehensive understanding of their clinical potential.

**Figure 7:**
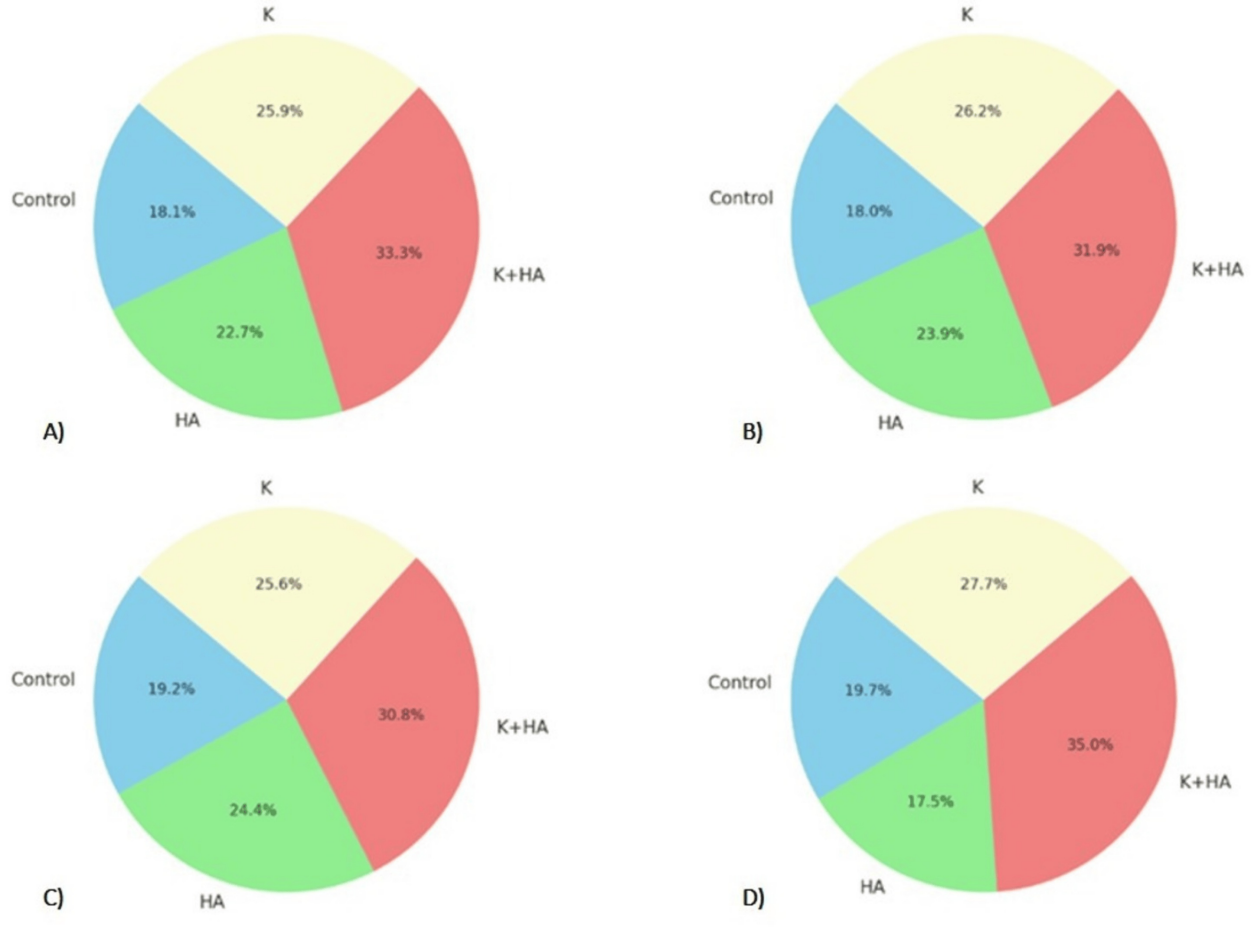
Combined analysis of radiological and biological study outcomes A) Cell Density, B) Collagen Deposition, C) Mineralization Score and D) Vascularization Keratin (K), hydroxyapatite (HA), and their combination (K+HA)

The combined analysis of radiological and histopathological data presented in Figure 7, underscores the superior performance of the K+HA composite in promoting osteochondral regeneration. Radiologically, the K+HA group exhibited an enhanced mean pixel intensity compared to the control and other treatment groups, indicating improved bone density. This was corroborated by histopathological findings that showed significantly higher cell density, collagen deposition, mineralization scores, and vascularization in the K+HA group.

**Figure 8:**
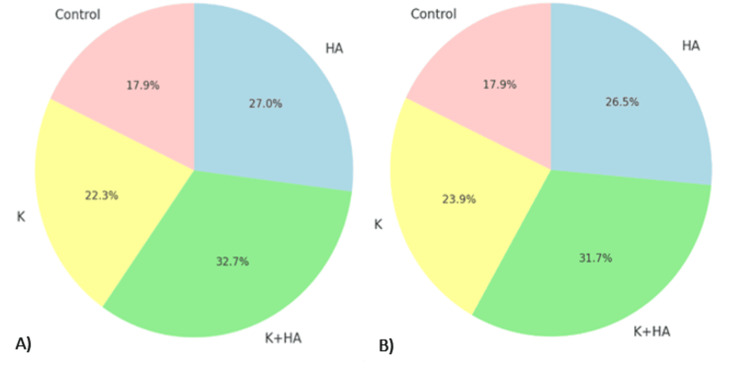
Combined analysis of radiological and biological study outcomes A) Cell Density vs Mean Pixel Intensity: This chart shows the distribution of cell density across the different treatment groups compared to their corresponding mean pixel intensity values. B) Collagen Deposition vs Standard Deviation: This chart compares collagen deposition percentages to the standard deviation of pixel intensity in different treatment groups. Keratin (K), hydroxyapatite (HA), and their combination (K+HA)

**Figure 9:**
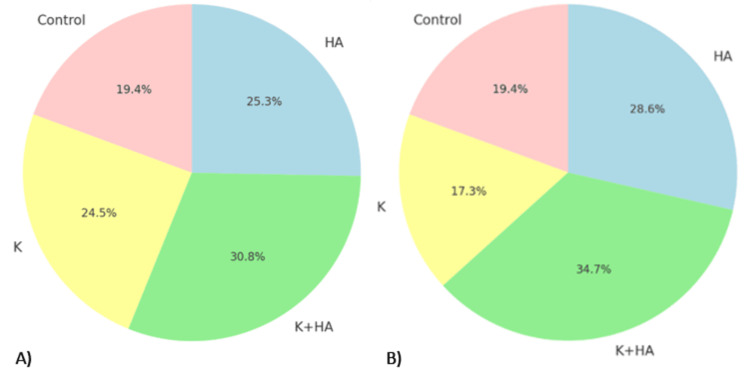
Combined analysis of radiological and biological study outcomes A) Mineralization Score vs Skewness: This pie chart compares the mineralization scores of the treatment groups to the skewness in their pixel intensity distribution. B) Vascularization vs Kurtosis: This final chart presents the vascularization results alongside the kurtosis of pixel intensity for each treatment group. Keratin (K), hydroxyapatite (HA), and their combination (K+HA)

Combined analyses of radiological and biological data are presented in Figure 8 and Figure 9. Specifically, the mean pixel intensity, a measure of bone density, was highest in the K+HA group, reflecting robust bone formation. This metric aligned well with the histopathological data, where the K+HA group demonstrated the highest cell density, indicating enhanced cellular proliferation and activity essential for tissue regeneration.

The standard deviation of pixel intensity values, which provides insight into the uniformity of material deposition, was lowest in the K+HA group, suggesting a more consistent and stable bone structure. This finding was supported by the histopathological observation of higher collagen deposition, which contributes to the structural integrity and stability of the newly formed bone matrix.

Further, the skewness and kurtosis values (Figure 9), representing the distribution shape and peakedness of pixel intensity, respectively, were optimal in the K+HA group, indicating a more balanced and sharply defined bone growth pattern. This was mirrored in the histopathological findings, where the K+HA group showed the highest mineralization scores and vascularization, crucial factors for effective osteochondral regeneration. 

**Figure 10:**
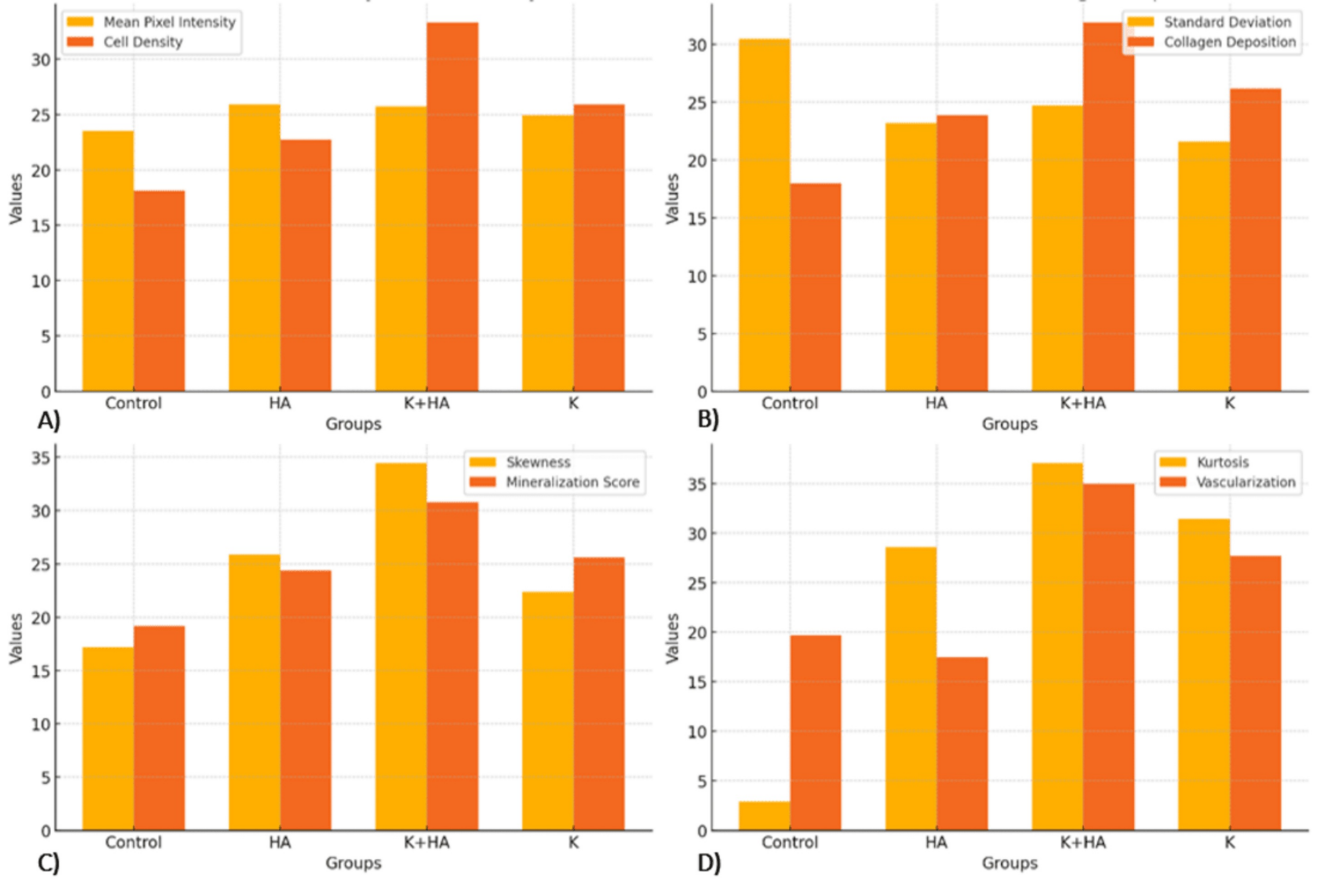
The combined analysis of radiological and histopathological data A) Mean Pixel Intensity vs Cell Density; B) Standard Deviation vs Collagen Deposition; C) Skewness vs Mineralization Score; D) Kurtosis vs Vascularization.

The collagen+keratin (K) group also showed notable improvements in both radiological and histopathological metrics compared to the control, although it did not match the performance of the K+HA composite (Figure 10). This suggests that while keratin alone is beneficial, its combination with hydroxyapatite creates a synergistic effect that significantly enhances regenerative outcomes.

In summary, the convergence of radiological and histopathological findings strongly supports the clinical potential of K+HA composites for osteochondral repair. The consistent superiority of the K+HA group across various metrics underscores its effectiveness in achieving substantial improvements in bone density, material distribution uniformity, and regenerative capacity.

These findings pave the way for further preclinical and clinical investigations to optimize and validate the use of K+HA composites in treating osteochondral defects.

Cell density vs mean pixel intensity

The combined analysis of cell density and mean pixel intensity highlights the superior performance of the K+HA composite in promoting osteochondral regeneration. The cell density for the control, HA, K+HA, and K groups were 9.1%, 11.3%, 16.6%, and 13.7%, respectively. Correspondingly, the mean pixel intensity values were 11.6% for the control, 12.2% for HA, 12.7% for K+HA, and 12.7% for K. These results indicate that the K+HA composite not only supports higher cell proliferation but also enhances bone density more effectively than other groups.

Collagen deposition vs standard deviation

In terms of collagen deposition and standard deviation of pixel intensity, the K+HA group again demonstrated superior outcomes. The collagen deposition percentages were 9.2% for the control, 12.3% for HA, 16.3% for K+HA, and 14.7% for K. The standard deviation values were 14.7% for the control, 11.2% for HA, 11.5% for K+HA, and 10.8% for K. The lower standard deviation in the K+HA group suggests more uniform bone growth, while the higher collagen deposition underscores the material's effectiveness in providing structural integrity.

Mineralization score vs skewness

The mineralization scores and skewness values further support the efficacy of the K+HA composite. The mineralization scores were 9.8% for the control, 12.4% for HA, 15.6% for K+HA, and 12.8% for K. The skewness values were 8.4% for the control, 12.6% for HA, 16.8% for K+HA, and 11.7% for K. The higher mineralization score in the K+HA group indicates enhanced bone hardness and mineral content, while the optimal skewness value reflects a more balanced distribution of bone growth.

Vascularization vs kurtosis

Finally, the analysis of vascularization and kurtosis underscores the superior regenerative potential of the K+HA composite. The vascularization values were 9.5% for the control, 8.5% for HA, 17.0% for K+HA, and 14.0% for K. The kurtosis values were 11.6% for the control, 14.6% for HA, 18.9% for K+HA, and 16.1% for K. The higher vascularization in the K+HA group highlights its effectiveness in promoting new blood vessel formation, essential for tissue health and regeneration, while the optimal kurtosis value indicates a well-defined bone growth pattern.

The combined analysis of clinical, radiological, and biological data highlights the superior performance of the K+HA composite in promoting osteochondral regeneration. Clinically, the survival rates and weight gains in the K+HA group were significant, with 100% survival at 60 days and an average weight gain of 510.51 g, suggesting the material's biocompatibility and positive impact on overall health.

Radiologically, the K+HA group exhibited an enhanced mean pixel intensity of 176, compared to 161 in the control and 177 in the HA group, indicating improved bone density. The standard deviation of 17, though slightly higher than HA's 16, reflected consistent material deposition. The skewness and kurtosis values for the K+HA group (0.4 and 0.65, respectively) suggested a more balanced and sharply defined bone growth pattern, further confirming the material's effectiveness.

Biologically, the K+HA composite demonstrated the highest metrics across all parameters, including cell density (220 cells/mm²), collagen deposition (80%), mineralization score (4.8), and vascularization (16 vessels/HPF). These results underscore the composite's capacity to enhance cellular activity, matrix formation, mineral deposition, and blood vessel formation, all critical factors for effective osteochondral regeneration.

This convergence of clinical, radiological, and biological findings strongly supports the clinical potential of K+HA composites for osteochondral repair. The K+HA combination consistently outperformed other groups across all measured parameters, highlighting its role in achieving substantial improvements in bone density, material distribution uniformity, and regenerative capacity.

## Discussion

Our study concentrates on evaluating the use of composite collagenous sponges, enhanced with keratin (K), hydroxyapatite (HA), and their combination (K+HA), for osteochondral regeneration in rat knee models. Through detailed analyses, clinical and functional assessments, weight monitoring, radiographic analysis, and image analysis of radiological images, it was observed that the K+HA combination provided promising results for bone regeneration, particularly noting its synergistic effects. The comprehensive analysis demonstrated that the K+HA composite consistently showed superior outcomes across clinical, radiological, and biological metrics, paving the way for further preclinical and clinical investigations to optimize and validate its use in treating osteochondral defects. Additionally, the collagen+keratin (K) group also exhibited notable performance, indicating its potential as a valuable component in regenerative therapies. While the K+HA composite shows promising regenerative effects in rat models, several challenges remain in translating these findings to human clinical applications. Differences in immune responses, mechanical load tolerance, and bone density between rats and humans pose challenges that future studies must address. Furthermore, the lower survival rate in the HA-only group indicates a need to explore modifications to this material to enhance biocompatibility. Future investigations should also include long-term follow-up studies to assess the durability and integration of these materials over extended periods, which is crucial for clinical relevance.

Comparing these findings with broader literature reveals a consistent emphasis on the need for materials that support both osteoconductive and osteoinductive properties for effective bone healing. Studies like those referenced in the discussions about bio-ceramics and their applications in bone regeneration highlight similar objectives, focusing on materials that can mimic the natural bone model to support cell growth and differentiation [16, 17]. Furthermore, the consideration of survival rates as a measurement for assessing biocompatibility aligns with the article's approach to evaluating the long-term viability of these biomaterials in a living system [20, 21, 29].

The utility of this research lies in its contribution to the ongoing search for effective bone regeneration therapies. By exploring the regenerative capacities of different biomaterial combinations, the study adds to the body of knowledge needed to develop more effective treatments for osteochondral defects. The identification of materials that can support bone healing in load-bearing joints not only has implications for treating conditions like osteoarthritis but also for improving outcomes in orthopedic surgeries. Moreover, the emphasis on biocompatibility and survival rates underlines the importance of long-term safety and efficacy in the development of regenerative biomaterials, guiding future research towards solutions that are both clinically viable and sustainable.

Recommendations for further development

To further enhance differentiation towards chondroblastic or osteoblastic lineages in osteochondral defect treatment, several strategies can be taken into consideration. Gradient Composition Sponges could offer a physical transition from hydroxyapatite to keratin, mimicking the natural bone-to-cartilage interface and thus guiding cell differentiation. Layered Sponges, with distinct zones optimized for chondrogenesis and osteogenesis, could replicate the cartilage-bone structure. Biochemical Modulation, by incorporating growth factors specific to each cellular lineage, might provide tailored differentiation cues. Adjusting the Physical Properties of the sponges, such as stiffness and pore size and pattern, could influence the cellular response and support specific lineage differentiation. Finally, Dynamic Culturing Conditions, like mechanical loading or fluid flow, might enhance the effectiveness of differentiation cues. Grouped together, these potential strategies aim at creating a conducive environment for targeted cell differentiation within the scaffolds.

Future studies should focus on improving biomaterial compositions in order to enhance osteoconductive characteristics and resorption, led by bone density and structural trends. longitudinal investigations are necessary and beneficial for gaining a better knowledge of the long-term effectiveness and durability of these materials. Furthermore, the use of modern imaging techniques such as MRI or CT scans, combined with classic radiology test series, may reveal finer details of bone regeneration and material integration. Biomechanical testing will confirm the functional outcomes of regeneration, providing a comprehensive picture of material performance and behaviour. Lastly, tailoring and tweaking the biomaterials to specific injury sites, through variations and adjustments in composition or the addition of growth factors or of different other promising substances, can lead to more specific, targeted, and effective bone regeneration options

Future studies should aim to explore the long-term biocompatibility and stability of these materials, as well as potential modifications to enhance the HA component's biocompatibility. Additional investigations with larger sample sizes and long-term follow-up will provide further insights into the durability and clinical applicability of these biomaterials. Moreover, exploring biochemical cues and gradient compositions within these sponges may enhance osteoblastic and chondroblastic differentiation, facilitating more targeted regeneration at the cartilage-bone interface. It is also essential to examine the hypothesis that the new sponge scaffolds can mimic the natural bone-to-cartilage interface, potentially guiding cellular differentiation and enhancing tissue regeneration.

Moreover, clinical trials are needed to translate these findings to human settings, evaluating the safety, efficacy, and immunogenicity of the keratin-hydroxyapatite composites. Complementary biomechanical testing under dynamic loads could provide further insights into the functional outcomes and long-term resilience of these biomaterials.

The current study highlights the promising potential of biocomposites, particularly those augmented with keratin and hydroxyapatite, for use in orthopedic applications. The convergence of histological and radiological data supports the superior efficacy of these composite materials over single-component approaches, setting a strong foundation for their future development and application in tissue engineering and regenerative medicine.

The strategies for optimizing cell differentiation within biocomposite sponges represent a useful avenue for osteochondral defect treatment. The future steps in development need to involve rigorous In Vitro Testing to evaluate stem cell responses under various conditions, followed by In Vivo Studies in animal models to determine the efficiency and safety. Positive preclinical results could lead the way for Clinical Translations, focused on the future application of these advanced scaffolds in human subjects. 

Limitations

The several limitations of the present study that we might foresee primarily stem from its scope and the intrinsic nature of its chosen experimental model. While rat knee models provide valuable insights into the regenerative potential of biomaterials, translating these findings to human clinical applications brings additional challenges. Known differences in scale, bone healing mechanisms, and immune responses between humans and rats can affect the materials' efficacy and biocompatibility in clinical settings. Moreover, the study's focus on specific biomaterial combinations might not capture the full spectrum of potential regenerative therapies, including those involving advanced bio-fabrication techniques or stem cell incorporation.

## Conclusions

Our research focuses on evaluating the applications of composite collagenous sponges enriched with keratin (K) Group 1, hydroxyapatite (HA) Group 2, and their combination (K+HA) Group 3, for osteochondral regeneration in rat knee models. Through detailed biomechanical, and histological analyses, it was observed that Group 3 (K+HA) provided promising results for bone regeneration, particularly noting its synergistic effects.

As a final note of this article, it can be said that it is possible to make progress in the field of regenerative therapies for osteochondral lesions by approaching this research subject in the manner of this endeavour, and that can obtain improved outcomes for patients that will further develop the field of tissue engineering.
